# Pilonidal Sinus Operations Performed Under Local Anesthesia versus the General Anesthesia: Clinical Trial Study

**DOI:** 10.5539/gjhs.v8n9p200

**Published:** 2016-01-22

**Authors:** Nasrin Rahmani, Afshin Gholipour Baradari, Seyed Mohammad-Javad Heydari Yazdi, Abolfazl Firouzian, Seyyed Abbas Hashemi, Mehran Fazli, Iman Sadeghian

**Affiliations:** 1 Department of General Surgery, School of Medicine, Mazandaran University of Medical Sciences, Sari, Iran; 2 Department of Anesthesia and Intensive Care, School of Medicine, Mazandaran University of Medical Sciences, Sari, Iran; 3 General Practitioner in Imam Khomeini Hospital of Esfarayen, Esfarayen Faculty of Medical Science, Esfarayen, Iran

**Keywords:** local, general, anesthesia, pilonidal sinus, operation

## Abstract

**Backgrounds:**

Various methods were defined to prepare patients for the pilonidal sinus surgery including local, spinal, and general anesthesia. But there is no powerful evidence to differ these procedures. Therefore, in the current study, we compared local and general anesthesia in the pilonidal sinus surgery.

**Methods and Material:**

In this clinical trial (IRCT201312031786N5) study 60 patients with the pilonidal sinus disease divided to two groups of local anesthesia versus general anesthesia. For local anesthesia we used 6ml of 2% lidocaine with an epinephrine (1:200,000), 6 ml of 0.5% bupivacaine, 1ml fentanyl (50 μg/ml), 1ml clonidine (75 μg/ml) and for general anesthesia fentanyl 1.5 μg.kg^-1^, thiopental 3-5 mg.kg^-1^, followed by the trachea intubation facilitated by atracurim 0.5 mg.kg-1 with maintenance of isoflurane 1-3% in nitrous oxygen 70% and oxygen 30%. The student t-test and Chi-square test were applied to evaluate the differences.

**Results:**

There were 30 patients with the mean age of 27.43±8.42 years in local anesthesia group and 30 cases with the mean age of 27.5±8.44 years underwent general anesthesia. The recovery time was significantly lower in the local anesthesia group (P=0.000). The oxygen saturation of the general anesthesia group was significantly higher at 1 and 20 minutes after the operation. The average of pain score was significantly higher in general anesthesia group at 3h and 6h after surgery (P<0.001). There were no significant differences in post-operative complications and hospital length of stay.

**Conclusion:**

This investigation revealed that local anesthesia has decreased pain during 48 hours after the surgery, shorter recovery time, and the less consumption of painkillers. So, we concluded that we can consider local anesthesia as a good alternative for the general anesthesia in the pilonidal sinus surgery.

## 1. Introduction

The pilonidal sinus has been defined as a chronic acquired inflammatory disease which occurs in the hair follicles in the buttock cleft at the bottom of the backbone (Muzi et al., 2010). Its etiology has not been well established, but the implantation of the loose hair into the depth of natal cleft which is increased between the buttocks, can cause the pilonidal sinus disease. The deep natal clefts are favorable environments for the sweating, maceration, bacterial contamination, and hair insertion. The causative factors of the pilonidal sinus are the nature of the hair itself, the force that cusses the hair insertion at the depth of the natal cleft and the vulnerability of skin Aydede, Erhan, Sakarya, & Kumkumoglu, 2001; McCallum et al., 2008; Al-Khamis, McCallum, King, & Bruce, 2010). This disease has a high incidence in young people. The incidence rate of the pilonidal sinus is 26 per 100000 population and it affects males 2.5 times more than females (Akca, Colak, Ustunsoy, Kanik & Aydin, 2005; Urhan, Kücükel, Topgul, Özer, & Sari, 2002). This disease is associated with a high morbidity and discomfort. It is also a cause of isolation from job and society which can result in a large number of the social and economic problems (Aldemir, Kara, Erten, & Taçyıldız, 2003).

There are several surgery methods for the pilonidal sinus disease (Ghnnam, & Hafez, 2011; J. Bascom & T. Bascom, 2002; Mentes et al., 2006; Cihan et al., 2006). One of these methods is primary closure (midline vs. off-midline). Those who are in favor of primary closure believe that the wounds heal more quickly after the primary closure than the open healing (McCallum, King, & Bruce, 2008). However, the surgeons have not reached to unanimity about the surgery as the best treatment for the pilonidal sinus disease (Ertan et al., 2005; McCallum, King, & Bruce, 2007).

To prepare patients for the pilonidal sinus surgery, different techniques of anesthesia may be used as local, spinal, and general anesthesia (H. Sungurtekin, U. Sungurtekin, & E. Erdem, 2003; Naja, Ziade, & El Rajab, 2003). Different health centers apply different anesthesia techniques and no unanimity decision has been reached about the best anesthesia technique (Khasawneh, Khamaiseh, & Kaabneh, 2005). Few studies have been done to compare the general and local anesthesia in primary midline closure for the pilonidal sinus disease and the results are contradictory (Sungurtekin et al., 2003; Naja, Ziade, & El-Rajab, 2003; Kayaalp et al., 2009). Thus this study was designed to make a comparison between the local and general anesthesia in the primary midline closure for the pilonidal sinus disease.

## 2. Materials and Methods

### 2.1 Ethics

All subjects gave their consent to participate in the study. This study was conducted in accordance with the Declaration of Helsinki and good clinical practice according to International Conference on Harmonisation guidelines. The ethics committee of Mazandaran Universtiy of Medical Sciences, Sari, IRAN approved this study.

### 2.2 Study Participants

**Figure 1:**
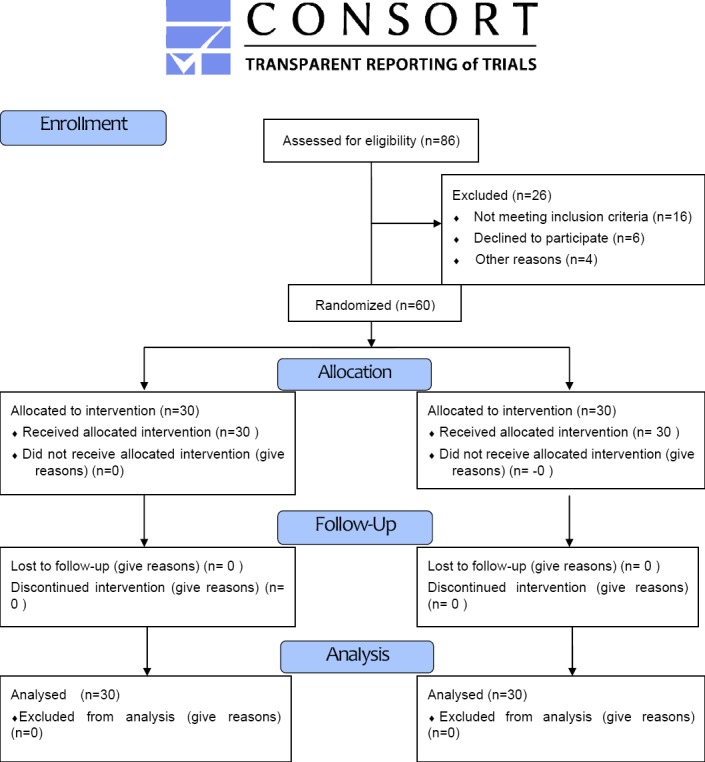
CONSORT 2010 Flow Diagram

This study is a randomized clinical trial (IRCT201312031786N5). In order to detect a 20% difference in the primary study endpoint with α=5% and ß=10%, a sample size of 30 patients in each group was found to be necessary for achieving the significant results (Sungurtekin et al., 2003; Naja, Ziade, & El Rajab, 2003) in Figure 1. All patients with the pilonidal sinus disease fulfilled the inclusion criteria. The exclusion criteria were the acute and recurrent pilonidal sinus disease, sensitivity to anesthesia drugs and the excessive overweight (BMI>35), diabetes mellitus, heart disease and the other underlying diseases (Kayaalp et al., 2009). Based on the inclusion and exclusion criteria, 60 patients with a confirmed disease were collected. Next, they were divided into two groups in a complete random way. One group received the local anesthesia (LA) and the other group received the general anesthesia (GA). The demographic data such as age, sex, height, and weight were recorded. All patients underwent the surgical excision with a primary midline closure. The patients who received 20 ml local anesthetic solution were put in a prone position. The injection was performed in the sacrococcygeal region around the pilonidal sinus and the anesthetic solution was a combination of 6ml of 2% lidocaine with an epinephrine (1:200,000), 6ml of 0.5% bupivacaine, 1ml fentanyl (50 μg/ml), 1ml clonidine (75 μg/ml) (Schmittner et al., 2013). If the patients needed sedation, the dose of 1.5-3 mg midazolam was given over a period of no less than 5 minutes (Kayaalp et al., 2009). The patients under the general anesthesia received the intravenous injection of the fentanyl 1.5 μg.kg^-1^, thiopental 3-5 mg.kg^-1^, followed by the trachea intubation facilitated by atracurim 0.5 mg.kg^-1^. Then, the patients were turned in the prone position and the anesthesia was maintained with an isoflurane 1-3% in nitrous oxygen 70% and oxygen 30%. At the end of the surgery, the patients were returned to the supine position and the residual neuromuscular blockade was antagonized with a neostigmine 0.05 mg.kg^-1^ and an atropine 0.01 mg.kg^-1^ (Søndenaa et al., 1995).

The amount of the intravenous midazolam, the total time on the operating room and the time for surgery for each patient were recoded. Then, in the recovery room, the patients’ number of breathing, oxygen saturation (monitoring with a pulse oximeter) and the blood pressure were recorded for every 10 minutes over 30 minutes after the surgery (monitoring started at the first minute). Also, the patients were asked whether they experienced any nausea, vomiting, and pain. Over next 30 minutes, the patients were asked about pain, nausea, vomiting, headache, and urinary retention (Kayaalp et al., 2009). The time passed from surgery to the first feeling of pain was recorded. The type and dose of analgesic used for each patient were recorded. In addition, the length of the hospital stay after the surgery was recoded. To measure the pain intensity, the visual analog scale was used and the patients were asked to rate their pain from 1-10 at 3,6,24,48, and 72 hours after the operation. All the patients were asked whether they were satisfied with the surgery procedure. All patients were seen after one week (Kayaalp et al., 2009).

### 2. Analytical Analysis

The student t-test was used to evaluate the difference in age, sex, height, weight, operation duration, recovery time, painkiller requirements, and the pain rate between two groups. Also, the Chi-square test was used to investigate the difference in age, postoperative nausea, vomiting, and length of hospital stay. A P value of <0.05 was considered as a significant.

### 3. Results

30 cases with the mean age of 27.43±8.42 years and 30 cases with the mean age of 27.5±8.44 years were assigned into LA and GA groups, respectively (P=0.97). In the LA group 19 cases (63.3%) and in the GA group 19 cases (63.3%) were male. The average of BMI was 25.04±3.12kg/m^2^ and 4.58±3.06kg/m^2^ for the LA and GA groups, respectively (P=0.56).

The average of the operation duration was 42.5±12.50 and 46.33±12.52 minutes for the LA and GA groups, respectively (P=0.24). The average of the recovery time was 18.83±5.03 and 14.16±3.95 minutes for the GA and LA groups, respectively. The recovery time of the LA group was significantly lower compared with the GA group (P=0.000). The vital signs of patients were recorded at 1, 10, 20, and 30 minutes following the operation and presented in Table 1. As shown in this table, there is no statistically significant difference in the blood pressure between the LA and GA groups (P>0.05). However, in the GA group the patients’ number of breathing at 1 (P=0.037), 10 (P=0.010) and 20(P=0.037) minutes following the surgery was higher compared with the LA group. But there was no statistically significant difference in the patients’ number of breathing at 30 minutes following the surgery between these 2 groups (P=0.067). A statistically significant difference was also observed in the oxygen saturation at 1 (0.026) and 20 (0.020) minutes following the surgery between these two groups. In fact, the oxygen saturation of the GA group was higher at 1 and 20 minutes following the surgery compared with the LA group. But there was no statistically significant difference in the oxygen saturation at 10(P=.30) and 30(P=0.10) minutes following the surgery between the LA and GA groups.

**Table 1:** the vital signs of patients at 1, 10, 20, and 30 minutes following the surgery

Vital signs	GA group	LA group	P value
Systolic blood pressure at 1 minute	119.06±10.65	119.96±6.77	0.07
Systolic blood pressure at 10 minutes	113.66±21.97	115.43±10.76	0.69
Systolic blood pressure at 20 minutes	117.16±10.31	115.36±7.56	0.44
Systolic blood pressure at 30 minutes	117.66±10.23	115.03±9.37	0.30
Diastolic blood pressure at 1 minute	75.50±9.29	76.33±5.34	0.67
Diastolic blood pressure at 10 minutes	74.70±8.50	73.86±8.72	0.70
Diastolic blood pressure at 20 minutes	76.33±8.60	74.16±7.55	0.30
Diastolic blood pressure at 30 minutes	74.23±8.13	76.36±7.55	0.29
Number of breathing at 1 minute	15.70±2.13	14.50±2.22	0.037
Number of breathing at 10 minutes	15.66±2.02	14.33±1.86	0.010
Number of breathing at 20 minutes	15.40±2.07	14.36±1.65	0.037
Number of breathing at 30 minutes	15.43±2.26	14.43±1.85	0.067
Oxygen saturation at 1 minute	98.86±1.56	97.93±1.59	0.026
Oxygen saturation at 10 minutes	98.53±1.99	98.03±1.71	0.30
Oxygen saturation at 20 minutes	98.96±1.27	98.13±1.43	0.020
Oxygen saturation at 30 minutes	98.96±1.15	98.43±1.30	0.10

The pain frequency and severity recorded following the operation and presented in Table 2. As shown in this table, frequency of pain in GA group was significantly higher in 3h, 6h, 24h, and 48h after surgery (P<0.001). Furthermore, the average of pain score was significantly higher in GA group at 3h and 6h after surgery (P<0.001).

**Table 2:** Frequency and average of pain after surgery

Evaluation Time		LA group	GA group	P vale
3 hours after surgery	mean±SD	1.88±1.21	3.36±1.56	0.002
	Frequency	17 (56.7%)	30 (100%)	<0.0001

6 hours after surgery	mean±SD	1.70±0.91	3.10±1.51	0.001
	Frequency	17 (56.7%)	30 (100%)	<0.0001

24 hours after surgery	mean±SD	2.14±0.90	2.54±1.71	0.55
	Frequency	7 (23.3%)	22 (73.3%)	<0.0001

48 hours after surgery	mean±SD	-	1.81±0.98	-
	Frequency	1 (3.3%)	10 (33.3%)	0.003

72 hours after surgery	mean±SD	-	-	-
	Frequency	0	3 (10%)	0.07

All patients who underwent LA received apotel injection while in the GA group, 19 patients (63.3%) received apotel injection, 10 patients (33.3%) received pethidine injection and 1 patient (3.3%) received diclofenac suppository. There was statistically significant difference in the prescribed painkillers between the LA and GA groups (P=0.002).

In terms of the postoperative complications, the wound infection occurred in 1 patient (3.3%) in the LA group while it was not observed in the GA group at all; there was no statistically significant difference between the LA and GA groups (P=0.33). Seroma occurred in 1 patient (3.3%) in the GA group while it was not observed in the LA group at all (0.33). The recurrence of disease only occurred in 1 patient (3.3%) in the GA group. Nausea occurred in 3 patients (10%) in the GA group while it was not observed in the LA group at all; no significant difference was observed between these 2 groups (P=0.076). Also, the urine retention was not seen at all. In general, the postoperative complications occurred in 6 patients (10%): 1 patient (3.3%) in the LA group and 5 patients (16.6%) in the GA group. No statistically significant difference was observed in the occurrence of the postoperative complications between the LA and GA groups (P=0.085).

The hospital stay was 1.03±0.50 and 1.23±0.77 days for the LA and GA groups, respectively. No statistically significant difference was observed in the hospital stay between the LA and GA groups (P=0.19).

The average of the time in return to work was 10.93±5.75 and 11.60±4.29 days for the LA and GA groups, respectively (P=0.61).

### 4. Discussion

Due to the fact that the pilonidal disease is usually seen in young adults and the estimated incidence is 26 per 100000 people, the selection of an appropriate method of surgery and anesthesia for reducing pain, recurrence, the operation duration and the length of the hospital stay is significant (Mentes et al., 2006; Søndenaa et al., 1995; Yalcin & Ergul, 2010). Thereby, in this study, all the 60 participants underwent a primary closure. The patients who undergo the primary closure, the duration of returning to the normal activity and the wound healing is shorter but the wound infection and recurrence is less compared with the open healing (McCallum, King, & Bruce, 2008). Several studies refer to the local and spinal anesthesia as alternative methods to the general anesthesia for the pilonidal sinus surgery (Schmittner et al., 2013). In this study, a comparison is made between the local and general anesthesia in the pain intensity, recovery time, the length of the hospital stay, and the complications.

Pain is one of the most effective factors which could affect the quality of patients’ life after a surgery. Many attempts have been done to select an appropriate surgery and anesthesia method to reduce the patients’ pain during and after the surgery (Yalcin, & Ergul, 2010; Møiniche et al., 2002). In this study, the patients in the LA group experienced less pain during 48 hours following the surgery compared with the patients in the GA group which was in concordance with the study of Naja et al. (Naja, Ziade, & El-Rajab, 2003) and Sungurtekin et al. (Sungurtekin et al., 2003). In this study, the most significant difference in the pain intensity was experienced during 4 hours following the surgery. Also, Tverskoy et al. conducted a study on the surgery of inguinal hernia using different anesthesia methods and reported that during 48 hours following the surgery the patients in the LA group experienced less pain compared with the GA group. But during the follow-up periods there was no difference between the LA and GA groups in the pain intensity (Tverskoy et al., 1990). In this study, the patients in the LA group required less consumption of painkillers compared with the GA group which was similar to the findings of Naja et al. and Tvverskoy et al. Besides, the less consumption of pain killers results in the reduction of cost and an avoidance of potential side effects of the painkillers (Sungurtekin et al., 2003; Naja, Ziade, & El Rajab, 2003; Tverskoy et al., 1990).

In this study, all the patients were put in the prone position. Although in the prone position the patients can feel constriction in their chest, it is reported that it does not have any negative effects on the respiratory mechanism. On the contrary, the prone positioning leads in the increased lung volume and the oxygenation of patients. Investing the vital signs in the LA and GA groups revealed that there was a difference between these 2 groups in the number of breathing which may be due to the use of different analgesic drugs (Pelosi et al., 1995).

In our study, the LA group’s length of the hospital stay was shorter compared with the GA group’s, but there was no statistically significant difference between them. All patients’ length of the hospital stay was at least 1 night. Naja et al. (2003) reported that the LA group’s length of the hospital stay was shorter compared with the GA group. In fact, 70% of the patients left hospital on the day of the surgery and more than two thirds of the patients in the GA group stayed for at least 1 night (H. Sungurtekin, U. Sungurtekin, & Erdem, 2003; Naja et al., 2003). This difference may be due to the different surgery procedures. More than 90% of the patients underwent an open healing. Our results revealed that the LA group’s recovery time was significantly shorter compared with the GA group’s which was in concordance with the study of Naja et al. (Yalcin, & Ergul, 2010) and Schmittner et al. (2013). Also, The LA group’s duration of returning to normal activity was shorter than the GA group’s; but there was no statistically significant difference between them.

### 5. Conclusion

This study revealed that administration of the local anesthesia for the pilonidal sinus surgery was associated with the decreased pain during 48 hours following the surgery, shorter recovery time, and the less consumption of painkillers. Therefore, the local anesthesia can be used as an appropriate substitute for the general anesthesia in the pilonidal sinus surgery.

## References

[1] Aldemir M, Kara İ H, Erten G, Taçyıldız İ (2003). Effectiveness of collagenase in the treatment of sacrococcygeal pilonidal sinus disease. Surgery today.

[2] Akca T, Colak T, Ustunsoy B, Kanik A, Aydin S (2005). Randomized clinical trial comparing primary closure with the Limberg flap in the treatment of primary sacrococcygeal pilonidal disease. British journal of surgery.

[3] Al-Khamis A, McCallum I, King P. M, Bruce J (2010). Healing by primary versus secondary intention after surgical treatment for pilonidal sinus. Cochrane Database Syst Rev.

[4] Aydede H, Erhan Y, Sakarya A, Kumkumoglu Y (2001). Comparison of three methods in surgical treatment of pilonidal disease. ANZ journal of surgery.

[5] Bascom J, Bascom T (2002). Failed pilonidal surgery: new paradigm and new operation leading to cures. Archives of Surgery.

[6] Cihan A, Ucan B. H, Comert M, Cesur A, Cakmak G. K, Tascilar O (2006). Superiority of asymmetric modified Limberg flap for surgical treatment of pilonidal disease. Diseases of the colon rectum.

[7] Ertan T, Koc M, Gocmen E, Aslar A. K, Keskek M, Kilic M (2005). Does technique alter quality of life after pilonidal sinus surgery?. The American journal of surgery.

[8] Ghnnam W. M, Hafez D. M (2011). Laser hair removal as adjunct to surgery for pilonidal sinus: Our initial experience. Journal of cutaneous and aesthetic surgery.

[9] Kayaalp C, Aydin C (2009). Review of phenol treatment in sacrococcygeal pilonidal disease. Techniques in coloproctology.

[10] Khasawneh M. A. S. S, Khamaiseh Q. M, Kaabneh A. B (2005). Local infiltration anesthesia versus spinal anesthesia in pilonidal sinus surgery. JRMS.

[11] McCallum I. J, King P. M, Bruce J (2008). Healing by primary closure versus open healing after surgery for pilonidal sinus: systematic review and meta-analysis. BMJ.

[12] McCallum I, King P. M, Bruce J (2007). Healing by primary versus secondary intention after surgical treatment for pilonidal sinus. Cochrane Database Syst Rev.

[13] Mentes O, Bagci M, Bilgin T, Coskun I, Ozgul O, Ozdemir M (2006). Management of pilonidal sinus disease with oblique excision and primary closure: Results of 493 patients. Diseases of the colon rectum.

[14] Møiniche S, Kehlet H, Dahl J. B (2002). A qualitative and quantitative systematic review of preemptive analgesia for postoperative pain relief: the role of timing of analgesia. Anesthesiology.

[15] Muzi M. G, Milito G, Cadeddu F, Nigro C, Andreoli F, Amabile D (2010). Randomized comparison of Limberg flap versus modified primary closure for the treatment of pilonidal disease. The American Journal of Surgery.

[16] Naja M, Ziade M, El-Rajab M (2003). Sacrococcygeal local anaesthesia versus general anaesthesia for pilonidal sinus surgery: A prospective randomised trial. Anaesthesia.

[17] Pelosi P, Croci M, Calappi E, Cerisara M, Mulazzi D, Vicardi P (1995). The prone positioning during general anesthesia minimally affects respiratory mechanics while improving functional residual capacity and increasing oxygen tension. Anesthesia Analgesia.

[18] Schmittner M. D, Dieterich S, Gebhardt V, Weiss C, Burmeister M. A, Bussen D. G (2013). Randomised clinical trial of pilonidal sinus operations performed in the prone position under spinal anaesthesia with hyperbaric bupivacaine 0.5% versus total intravenous anaesthesia. International journal of colorectal disease.

[19] Søndenaa K, Andersen E, Nesvik I, Søreide J (1995). Patient characteristics and symptoms in chronic pilonidal sinus disease. International journal of colorectal disease.

[20] Sungurtekin H, Sungurtekin U, Erdem E (2003). Local anesthesia and midazolam versus spinal anesthesia in ambulatory pilonidal surgery. Journal of clinical anesthesia.

[21] Tverskoy M, Cozacov C, Ayache M, Bradley E. L, Kissin I (1990). Postoperative pain after inguinal herniorrhaphy with different types of anesthesia. Anesthesia Analgesia.

[22] Urhan M. K, Kücükel F, Topgul K, Özer İ, Sari S (2002). Rhomboid excision and Limberg flap for managing pilonidal sinus. Diseases of the colon rectum.

[23] Yalcin S, Ergul E (2010). A single-surgeon, single-institute experience of 59 sinotomies for sacrococcygeal pilonidal disease under local anesthesia. Bratisl Lek Listy.

